# Molecular determinants of proton selectivity and gating in the red-light activated channelrhodopsin Chrimson

**DOI:** 10.1038/s41598-017-09600-8

**Published:** 2017-08-30

**Authors:** Johannes Vierock, Christiane Grimm, Noam Nitzan, Peter Hegemann

**Affiliations:** 1 Institute of Biology, Experimental Biophysics, Humboldt-Universität zu Berlin, 10115 Berlin, Germany; 2 Present Address: Neuroscience Research Center, Charité - Universitätsmedizin Berlin, Charitéplatz 1, 10117 Berlin, Germany

## Abstract

Channelrhodopsins are light-gated ion channels of green algae used for the precise temporal and spatial control of transmembrane ion fluxes. The channelrhodopsin Chrimson from *Chlamydomonas noctigama* allows unprecedented deep tissue penetration due to peak absorption at 590 nm. We demonstrate by electrophysiological recordings and imaging techniques that Chrimson is highly proton selective causing intracellular acidification in HEK cells that is responsible for slow photocurrent decline during prolonged illumination. We localized molecular determinants of both high proton selectivity and red light activation to the extracellular pore. Whereas exchange of Glu143 only drops proton conductance and generates an operational Na-channel with 590 nm activation, exchange of Glu139 in addition increased the open state lifetime and shifted the absorption hypsochromic by 70 nm. In conjunction with Glu300 in the center and Glu124 and Glu125 at the intracellular end of the pore, Glu139 contributes to a delocalized activation gate and stabilizes by long-range interaction counterion configuration involving protonation of Glu165 that we identified as a key determinant of the large opsin shift in Chrimson.

## Introduction

Understanding the complex interplay of molecular processes in living systems requires the ability to control the activity of distinct molecular components in a non-invasive manner. In optogenetics, the expression of natural or engineered photoreceptors in a genetically defined cell type enables modulation of molecular events such as changes in second messenger concentration, enzymatic activities or transmembrane ion fluxes by light^1–3^. Especially microbial rhodopsins are highly versatile tools for the rapid and repetitive manipulation of membrane voltage^4^. In contrast to prokaryotic proton and chloride pumps, channelrhodopsins (ChR) trigger and inhibit action potentials under moderate light intensities by conducting either cations or chloride into the cell depending on the ChR variant^5–7^.

Recent studies have likewise emphasized the impact of microbial rhodopsin stimulation on local sodium, calcium, chloride, or proton concentrations leading to numerous secondary effects at the subcellular level^8^. These effects include the active import of calcium ions into astrocytes by a Na^+^-Ca^2+^-exchanger^9^, the pH-induced influx of calcium in synaptic terminals^10^, shifts in the gamma-aminobutyric acid (GABA) receptor reversal potential in synaptic terminals^11^, or the activation of acid-sensing ion channels (ASIC) in proton microdomains on the extracellular surface^12^ or near astrocytes expressing proton pumps^13^. Therefore, knowing the exact nature of the transported ion is critical for the interpretation of optogenetic experiments. Further, to better understand the functional impact of local ion concentration changes in neurons and subcellular compartments, ChRs with a distinct preference for a specific ion are of major interest.

To date, our understanding of ChRs mainly stems from Channelrhodopsin 2 of *Chlamydomonas reinhardtii* (*Cr*ChR2)^14^ and its chimeric derivative C1C2^15^. After absorbing blue light, ChRs undergo a cyclic sequence of conformational changes involving all-*trans* to 13-*cis* isomerization of the retinal chromophore^16, 17^, deprotonation of the retinal Schiff-base^18^, an outward tilt of helix 2 and helix 7^19, 20^, and the subsequent hydration and opening of the ion conducting pore formed by helices 1, 2, 3, and 7 on a nano- to millisecond timescale^21–23^. The putative ion-conducting pore is flanked by a periodic sequence of five conserved glutamates in helix 2 which are referred to as E1’ to E5’ and are numbered from the intracellular to the extracellular side of the protein (E82, E83, E90, E97, and E101 in *Cr*ChR2). The extracellular glutamates E4’ and E5’ contribute to an open access channel that is separated from the cytoplasm by two hydrophobic constrictions that are close to the retinal Schiff base and to the intracellular end of the pore, termed the central and inner gate, respectively. In the central gate, E3’ contributes to a hydrogen bonding network involving S63 and N258, which interconnects helices 1, 2, and 7, whereas in the inner gate, E1’ and E2’ are engaged in hydrogen bonds to H134 and R268 in helix 3 and helix 7^24^.

Especially the central gate residue E3’ is reportedly a key determinant for gating and ion selectivity in *Cr*ChR2. In the E90-Helix2-Tilt model^21^, retinal isomerization induces a displacement of N258 via the protein backbone, causing a reduction in the number of hydrogen bonds between E3’ and N258. Consequently, E3’ becomes more flexible, flips outward, deprotonates, and is finally stabilized by the adjacent K93, which causes an outward tilt of helix 2 that allows water to invade the ion-conducting pore through the access channel. After initial transitions at the central gate and water influx from the extracellular side, destabilization of the inner gate salt bridges between E2’ and R268 as well as E3’ and H134 further impairs interaction of helixes 2, 3, and 7 and prepares the final opening of the pore by a second gating transition. Replacing E3’ with a polar glutamine or a neutral alanine residue reduces proton conductance stepwise^25, 26^, whereas replacing E3’ with a positively charged arginine abolishes cation conductance, transforming *Cr*ChR2 into an anion-selective ChR^6^.

A long-term goal in optogenetic engineering was the design of ChR variants that absorb red light allowing deeper tissue penetration in *in-vivo* experiments and the independent activation of cells expressing different ChRs. The molecular engineering of the ChR chimeras C1V1^27^ and ReaChR^28^ has already reduced the problem of light scattering in neuronal tissues by shifting the peak sensitivity to green or yellow light; however, a combination with blue light-sensitive ChRs remains challenging due to a strong overlap in the action spectra. In an extended screen for photocurrents of 61 potential ChRs in human embryonic kidney (HEK) cells, Klapoetke *et al*. discovered *Cn*ChR1, a red-light activated ChR from the freshwater algae *Chlamydomonas noctigama*, which the authors named Chrimson^29^.

Chrimson exhibits maximal photocurrents at 585 nm, which is red-shifted more than 40 nm compared with all other ChRs. Due to its red-shifted peak activity, a combination of Chrimson and Chronos, a blue light-sensitive ChR, enabled the dual color activation of independent neuronal populations using blue and red light, respectively^29^. Because Chrimson can be activated even by intense far-red light up to 720 nm, a wavelength that is distinct from the light used for other optogenetic actuators or reporters including GCaMP^30, 31^ and is beyond the operational range of many natural photoreceptor systems such as *Drosophila* rhodopsins^32^, Chrimson was quickly utilized for optogenetic and behavioral studies in *Caenorhabditis elegans* ^33^ and *Drosophila melanogaster* ^34, 35^.

Due to its high potential for optogenetic applications, we subjected Chrimson to a detailed electrophysiological characterization and compared its ion selectivity with other ChRs. We demonstrate that under physiological conditions, Chrimson photocurrents are primarily carried by protons, which causes significant intracellular acidification in HEK cells during prolonged illumination. We identified an altered gate structure in Chrimson compared to other channelrhodopsins featuring an additional putative pore constriction around E4’, that determines proton selectivity, photocurrent kinetics, and is essential for the large opsin shift in Chrimson.

## Results

### Chrimson photocurrents are highly pH dependent and proton selective

**Figure 1:**
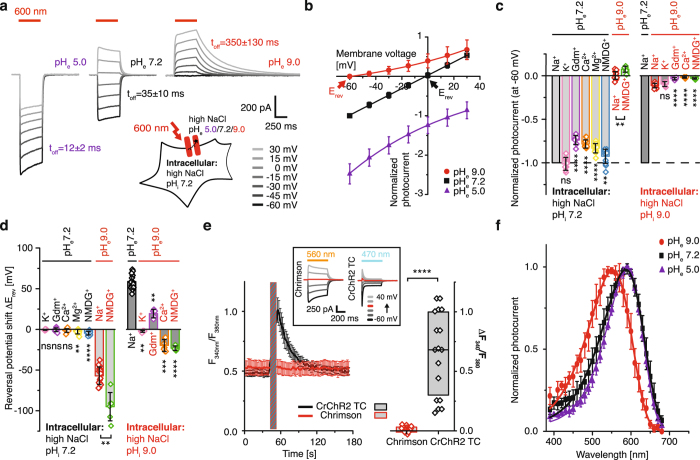
pH dependence and ion selectivity of Chrimson photocurrents. (**a**) Chrimson photocurrent traces measured in HEK293 cells during illumination at 600 nm (2.37 mW × mm^−2^) at different voltages and different extracellular pH_e_ with symmetric intra- and extracellular 110 mM NaCl and intracellular pH_i_ 7.2 (**b**) Current-voltage dependence of normalized peak photocurrents at different extracellular pH_e_ as represented in (**a**) (mean ± SD; pH_e_ 5: purple, n = 8 cells; pH_e_ 7.2: black, n = 37 cells; pH_e_ 9.0: red, n = 16 cells) (**c**) Normalized photocurrent amplitudes at −60 mV and different extracellular ionic conditions with two different intracellular solutions of 110 mM NaCl pH_i_ 7.2 (left) and 110 mM NaCl pH_i_ 9.0 (right) (mean ± SD; LJP corrected; normalized to extracellular 110 mM NaCl, pH_e_ 7.2 (dashed line) t-test for comparison to symmetric ionic conditions for both intracellular conditions respectively; 110 mM KCl pH_e_ 7.2, n = 8, p = 0.7; 110 mM GdmCl pH_e_ 7.2, n = 8, p < 0.0001; 55 mM CaCl_2_ pH_e_ 7.2, n = 12, p < 0.0001; 55 mM MgCl_2_ pH_e_ 7.2, n = 12, p < 0.0001; 110 mM NMDGCl pH_e_ 7.2, n = 14, p = 0.004; 110 mM NaCl pH_e_ 9.0/pH_i_ 7.2, n = 16, p < 0.0001; 110 mM NMDGCl pH_e_ 9.0/pH_i_ 7.2, n = 6, p < 0.0001/0.002; 110 mM NaCl pH_e_ 9.0/pH_i_ 9.0, n = 17; 110 mM KCl pH_e_ 9.0, n = 5, p = 0.3; 110 mM GdmCl pH_e_ 9.0, n = 5, p < 0.0001; 55 mM CaCl_2_ pH_e_ 9.0, n = 7, p < 0.0001; 110 mM NMDGCl pH_e_ 9.0, n = 6, p < 0.0001) **(d)** Reversal potential shifts with two different intracellular solutions of 110 mM NaCl pH_i_ 7.2 (left) and 110 mM NaCl pH_i_ 9.0 (right) compared to symmetric conditions of either extracellular 110 mM NaCl and pH_e_ 7.2 (left) or extracellular 110 mM NaCl and pH_e_ 9.0 (right) (Mean ± SD, LJP corrected, t-test for comparison to symmetric ionic conditions for both intracellular conditions respectively; 110 mM KCl pH_e_ 7.2, n = 8, p = 0.05; 110 mM GdmCl pH_e_ 7.2, n = 8, p = 0.6; 55 mM CaCl_2_ pH_e_ 7.2, n = 12, p = 0.06; 55 mM MgCl_2_ pH_e_ 7.2, n = 12, p = 0.005; 110 mM NMDGCl pH_e_ 7.2, n = 14, p < 0.0001; 110 mM NaCl pH_e_ 9.0/pH_i_ 7.2, n = 16, p < 0.0001; 110 mM NMDGCl pH_e_ 9.0/pH_i_ 7.2, n = 6, p < 0.0001/0.002; 110 mM NaCl pH_e_ 7.2/pH_i_ 9.0, n = ; 110 mM KCl pH_e_ 9.0, n = 5, p = 0.005; 110 mM GdmCl pH_e_ 9.0, n = 5, p = 0.001; 55 mM CaCl_2_ pH_e_ 9.0, n = 7, p = 0.0004; 110 mM NMDGCl pH_e_ 9.0, n = 6, p < 0.0001)) **(e)** Fluorescence ratio *F*_340_/*F*_380_ (left: mean ± SE) and corresponding changes Δ(*F*_340_/*F*_380_) (right: mean ± SD, 0.02 s after activation, two-sample t-test p < 0.0001) of Fura-2 loaded HEK293 cells after excitation at 340 nm and 380 nm following the activation of *Cr*ChR2 T159C at 450 nm (n = 17 cells) or Chrimson at 560 nm (n = 30 cells) for 10 s in an extracellular solution of 70 mM CaCl_2_. Inlet: Photocurrents of both ChRs measured under the same conditions. (**f**) Chrimson action spectra after 10 ms excitation at different wavelength of equal photon count (Mean ± SD, pH_e_ 5.0: purple, −60 mV, n = 6 cells; pH_e_ 7.2: black, −60 mV, n = 9 cells; pH_e_ 9.0: red, +30 mV, n = 4 cells).

Chrimson was fused at the C-terminus to the monomeric cerulean fluorescent protein mCerulean3.0 and expressed in HEK cells in three different targeting variants that differ in their N-terminal sequence with minor consequences on overall photocurrent properties (Supplementary Fig. S1a–g). Under continuous high-intensity orange light and pH_i/e_ 7.2, Chrimson displayed robust photocurrents that reached half-maximal amplitude in less than 4 milliseconds and varied in size and direction depending on the applied voltage (Fig. 1a). Unlike other ChRs, Chrimson did not exhibit a transient peak that rapidly decayed to a stationary level at continuous illumination but instead displayed gradual slow photocurrent decline that largely depended on channel expression and intracellular buffer concentration (Supplementary Fig. S4d+e). After the light source was turned off, the photocurrents decayed in a biexponential manner on a millisecond time scale. For simplification we summarized closing kinetics in an apparent time constant of channel closure (τ_app,off_) that was highly pH dependent - increasing by a factor of 30 between pH_e_ 5 and pH_e_ 9.0﻿ - and voltage dependent at neutral and alkaline pH_e_ (Supplementary Fig. S2a). The photocurrent-voltage relationship revealed that in contrast to all previously characterized ChRs, Chrimson did not exhibit voltage-dependent rectification under symmetric conditions (Fig. 1b). Most strikingly, despite symmetric NaCl concentrations, variation in the extracellular proton concentration resulted in large reversal potential shifts and photocurrents only inward or outward directed in the analyzed voltage range, indicating a high proton contribution to overall photocurrents (Fig. 1a,b).

In order to investigate the contribution of protons and other cations to the overall photocurrent in further detail, we exchanged extracellular sodium versus other mono- and divalent ions such as potassium, calcium, magnesium and the small and large organic cations guanidinium (Gdm^+^) and N-Methyl-D-glucamine (NMDG^+^), which are well and not conducted in *Cr*ChR2^14^. In Chrimson reduction of extracellular protons to pH_e_ 9.0 resulted in a dramatic attenuation of photocurrent amplitude (Fig. 1c left) and a large reversal potential shift (Fig. 1d left). In contrast, reduction of extracellular sodium by substitution with the non-conducted NMDG^+^ caused only a small reversal potential shift and reduced inward photocurrents by less than ten percent confirming that more than 90% of charges were carried by protons for Chrimson photocurrents at pH_e_ 7.2. Interestingly, substituting extracellular sodium with the small organic cation guanidinium or the divalent ions calcium and magnesium reduced photocurrent amplitude even further than NMDG^+^ whereas the reversal potential was nearly unchanged, indicating an overall blockage of photocurrents by guanidinium and divalent ions. At reduced intra- and extracellular proton concentrations negative reversal potential shifts for sodium substitution with calcium and positive shifts for substitution with guanidinium show that - although again both ions reduce photocurrent amplitudes by blocking the passage of other ions - guanidinium permeates Chrimson at a really low rate whereas calcium is not conducted (Fig. 1d right, Supplementary Fig. S3).

In order to confirm the lack of calcium permeation through Chrimson, HEK cells were loaded with Fura-2 acetoxymethyl (AM) ester, which serves as a ratiometric Ca-sensitive fluorescent dye. Blue illumination of *Cr*ChR2-T159C-expressing cells for 15 s at a 70 mM extracellular calcium concentration caused a significant change in Fura-2 fluorescence, representing an increase in intracellular calcium that returned to the baseline level within seconds when illumination was terminated (Fig. 1e, left). In contrast, the illumination of Chrimson-expressing cells with orange light caused no observable increase in intracellular calcium (Fig. 1e). Under these conditions, both ChRs exhibited equivalent peak photocurrents, whereas the overall ion influx in *Cr*ChR2-T159C was smaller due to the more pronounced inactivation as shown in a separate electrical experiment (Fig. 1e, inset).

Considering the high pH dependence of photocurrent kinetics, we wondered if the protonation of critical residues inside the protein might influence the spectral photocurrent properties of Chrimson as previously reported for *Cr*ChR1 and *Vc*ChR1^36, 37^. We measured photocurrent action spectra for 10 ms flashes of different light colors but equal photon flux and observed a 35 nm blue shift at pH_e_ 9.0 compared to pH_e_ 7.2 (Fig. 1f).

### Intracellular acidification causes the slow photocurrent decline

**Figure 2:**
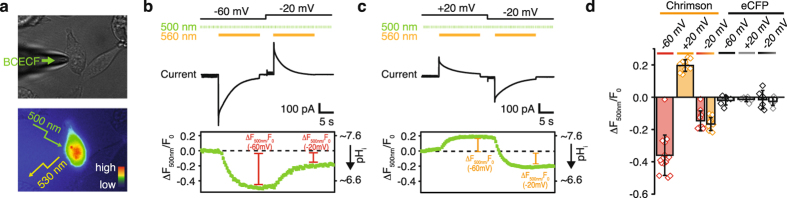
Intracellular acidification and slow photocurrent decline. (**a**) Experimental scheme of simultaneous voltage-clamp and intracellular pH measurements: HEK293 cells were loaded with BCECF free acid using the patch pipette. pH-dependent fluorescence of BCECF at 530 nm was excited by 5 ms pulses of 500 nm light during electrical recordings (bright-field and fluorescent images of a transfected HEK293 cell in the whole-cell configuration. The fluorescence intensity is represented by false colors). (**b**) Top: voltage and illumination protocol featuring two successive 15 s illuminations of 560 nm first at −60 mV and then at −20 mV and continuously pulsed excitation of BCECF at 500 nm. Middle: Photocurrent recording in symmetric 110 mM NaCl and pH_e/i_ 7.2. Bottom: Normalized BCECF fluorescence and the corresponding approximated intracellular pH_i_ values estimated by the BCECF calibration in supplementary Fig. 3b. (**c**) The same recording as in (**b**) with a cell first clamped at +20 mV and then at −20 mV. (**d**) The normalized changes in intracellular fluorescence (with approximated intracellular pH_i_ values) after 560 nm illumination at −60 mV (red) or +20 mV (orange) and after the second illumination at −20 mV (red/orange) in Chrimson or enhanced cyan fluorescent protein (gray) expressing cells as a control (mean ± SD, Chrimson n = 10–15 cells, eCFP n = 6 cells).

Because it was conceivable that prolonged illumination at negative holding potential causes acidification of the intracellular bulk phase and consequently disfavors inward currents we performed pH_i_ imaging in parallel with electrical recordings. During patch clamp recordings the intracellular pH_i_ can be deduced from fluorescence changes ΔF_500_/F_0_ of the soluble pH indicator BCECF that we calibrated using the H^+^/K^+^ exchanging ionophore nigericin (Supplementary Fig. S4a+b). We supplied BCECF through the patch pipette and illuminated the cell for 15 s at −60 mV or +20 mV (Fig. 2a–c). The observed fluorescence changes paralleled the recorded photocurrent decline and leveled off at intracellular pH_i_ 6.6 after proton influx at −60 mV and at pH_i_ 7.6 after proton efflux at +20 mV (Fig. 2d). Afterwards membrane voltage was changed to −20 mV in both cases (close to reversal potential) and the cells were illuminated again. As expected due to intracellular pH changes the photocurrents were now outward directed after pre-illumination at −60 mV (Fig. 2b) and inward directed after pre-illumination at +20 mV (Fig. 2c) but rapidly declined to a small inward current in both cases. Likewise also the BCECF fluorescence changed and reached a final pH_i_ 7.0 independent of the preceding experiments. The photocurrent decline during illumination and the reversal potential shifts were less pronounced or even abolished at either small photocurrent amplitudes or higher internal buffer concentration, confirming intracellular pH changes as the cause of slow photocurrent decline under prolonged illumination (Supplementary Fig. S4c–e).

### Chrimson is more proton selective than other ChRs

**Figure 3:**
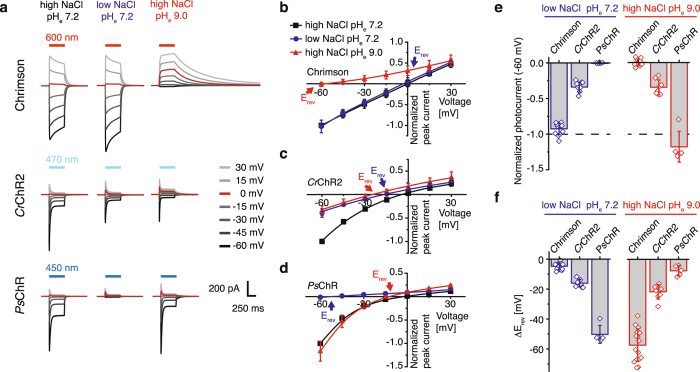
Comparison of Chrimson with other ChRs. (**a**) Representative photocurrents of Chrimson, *Cr*ChR2 and *Ps*ChR at different voltages and symmetric solution of 110 mM NaCl and pH_e_ 7.2 (left), after reduction of extracellular sodium (1 mM NaCl and pH_e_ 7.2, middle) and proton concentration (110 mM NaCl, pH_e_ 9.0 right) (**b**) The current-voltage dependence of normalized peak photocurrents of Chrimson under the ionic conditions described in (a) (mean ± SD; black: symmetric 110 mM NaCl and pH_i/e_ 7.2, n = 37 cells; blue: 1 mM NaCl and pH_e_ 7.2, n = 14 cells; red: 110 mM NaCl and pH_e_ 9.0, n = 16 cells). **(c)** Normalized peak photocurrents of *Cr*ChR2 plotted against the applied membrane voltage (mean ± SD; n = 8–12 cells). **(d)** Normalized peak photocurrents of *Ps*ChR plotted against the applied membrane voltage (mean ± SD; n = 5 cells). **(e)** Photocurrent amplitudes at −60 mV normalized to symmetric standard conditions of 110 mM NaCl pH_e_ 7.2 (dashed line) corresponding to (**a**), (Mean ± SD; LJP corrected; blue: 1 mM NaCl and pH_e_ 7.2; red: 110 mM NaCl and pH_e_ 9.0; intracellular solution: 110 mM NaCl and pH_i_ 7.2; Chrimson n = 14–16, *Cr*ChR2 n = 8–9, *Ps*ChR n = 5) (**f**) Corresponding reversal potential shifts upon extracellular buffer exchange from symmetric 110 mM NaCl and pH_e_ 7.2 to low extracellular sodium (left, blue, 1 mM NaCl and pH_e_ 7.2) or low extracellular proton (right, red, 110 mM NaCl and pH_e_ 9.0) concentration (mean ± SD; LJP corrected; Chrimson n = 14–16, *Cr*ChR2 n = 8-9, *Ps*ChR n = 5).

For better classification among ChRs, the proton versus sodium conductance of Chrimson was compared with *Cr*ChR2 and the sodium selective channelrhodopsin *Ps*ChR from *Platymonas tetraselmis subcordiformis* ^38^. Using symmetric sodium and proton concentrations, we measured the photocurrents for all constructs at different voltages and then either removed extracellular sodium (110 mM NaCl to 1 mM NaCl by substitution with the non-conducted NMDGCl) or protons (pH_e_ 7.2 to pH_e_ 9.0; Fig. 3a). Whereas upon reduction of extracellular Na^+^ Chrimson photocurrents barely changed (Fig. 3a+b), photocurrents of *Cr*ChR2 were significantly decreased in amplitude or even completely disappeared in case of *Ps*ChR (Fig. 3a,c,d and e). Accordingly, at high extracellular sodium and pH_e_ 9.0, the inward currents of *Ps*ChR and *Cr*ChR2 remained high whereas they nearly disappeared with Chrimson. In line, reduction of the extracellular sodium caused larger *E*_rev_ shifts in *Ps*ChR and *Cr*ChR2 compared to Chrimson (Fig. 3f) whereas *E*_rev_ changes after reducing extracellular proton concentrations were most pronounced in Chrimson. Assuming that only monovalent ions and protons are conducted, proton selectivity near *E*_rev_ can be quantified by the Goldmann-Hodgkin-Katz voltage equation. The relative permeability of protons in Chrimson (P_H+_/P_Na+_ = 1.3 ± 0.4 *10^7^) surpasses the permeability ratio of *Cr*ChR2 (P_H+_/P_Na+_ = 2.2 ± 0.3 *10^6^) and *Ps*ChR (P_H+_/P_Na+_ = 4.3 ± 1.8 *10^5^) by far.

### Amino acid substitution along the ion conducting pathway are crucial for channel gating and opsin shift

**Figure 4:**
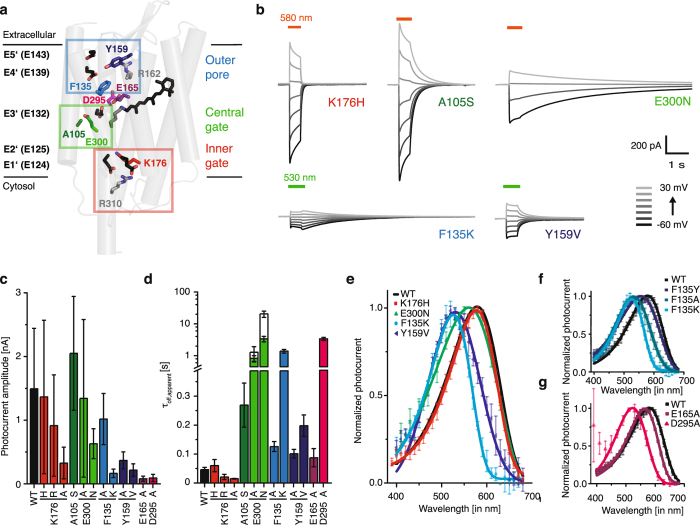
Mutation analysis of substitutions along the ion conducting pathway. (**a**) Homology model of Chrimson (based on C1C2 4yzi.pdb) with pore lining glutamates E1’ to E5’ (in black) and amino acid substitutions compared to C1C2 in the inner gate (red), central gate (green) and the outer pore (blue) (**b**) Representative photocurrents of Chrimson mutants (incorporating one-by-one the corresponding residues of C1C2) at different voltages in symmetric 110 mM NaCl and pH_i,e_ 7.2 (**c**) Photocurrent amplitudes of WT and pore mutants at −60 mV and symmetric 110 mM NaCl and pH_e,i_ 7.2 (mean ± SD; WT n = 16 cells; K176H n = 10, K176R n = 14, K176A n = 8, A105S n = 16, E300A n = 8, E300N n = 10, F135A = 18, F135K = 18, Y159A n = 6, Y159V n = 7, E165A n = 7, D295A n = 5). (**d**) Apparent off-kinetics at −60 mV τ_apparent,off,−60mV_ of Chrimson WT and pore mutants at pH_e,i_ 7.2. Empty columns for E300A and E300N represent conductance measurements by short 20 ms voltage pulses to −60 mV at 0.5 Hz and a holding potential of 0 mV for the reduction of current decline due to intracellular acidification (mean ± SD; WT n = 18 cells; K176H n = 9, K176R n = 7, K176A n = 8, A105S n = 13, E300A n = 8/2, E300N n = 10/8, F135A = 18, F135K = 17, Y159A n = 6, Y159V n = 7, E165A n = 7, D295A n = 5) (**e**) Normalized peak photocurrents after 10 ms excitation at different wavelengths of equal photon count for selected mutants (mean ± SD; symmetric 110 mM NaCl, pH_e,i_ 7.2 and −60 mV; WT n = 10 cells; K176H n = 7, E300N n = 8, F135K = 11, Y159V n = 7), (**f**) different mutants of F135 (mean ± SD; WT n = 10, F135K n = 11, F135Y n = 7, F135A n = 15) (**g**) and of putative counter ion mutants (mean ± SD; WT n = 10, E165 n = 11, D295 n = 7).

In comparison to *Cr*ChR2, Chrimson features important amino acid substitutions along the putative ion-conducting pathway (Fig. 4a, Supplementary Fig. S5). Both channel gates of *Cr*ChR2 are substantially changed due to the substitution of H134 in the inner gate and S63 and N258 in the central gate by K176, A105 and E300 in Chrimson. Also in the outer pore the two aromatic residues F135 and Y159 replace K93 in vicinity to counterion complex and N117 in the extracellular access channel. We mutated each of these positions (Fig. 4b) and analyzed photocurrent amplitude (Fig. 4c), kinetics (Fig. 4d) and wavelength dependence (Fig. 4e).

Mutation of the inner gate K176 in Chrimson accelerates photocurrent off kinetics– most dramatically for K176A - (Fig. 4d) and reduces voltage and pH_e_ dependence of channel closing rates (Supplementary Fig. S6a). In contrast mutation of A105 and E300 decelerated closing kinetics (Fig. 4d). Determination of channel closing kinetics was particularly challenging for the highly conductive mutant E300N since both channel closing as well as intracellular acidification contribute to the decline of photocurrents. Clamping the illuminated cell to 0 mV (reversal potential) and only probing channel conductance by repetitive short voltage pulses yielded ten fold larger closing kinetics for E300N and revealed that channel closure of E300N was 20 times slower compared to E300A (Fig. 4d empty bars). Also mutation of both outer pore residues F135 and Y159 decelerated closing kinetics, but furthermore reduced photocurrent amplitudes and most importantly caused large hypsochromic shifts of up to 70 nm in spectral sensitivity (Fig. 4e). The large blue shift of F135 was essentially based on elimination of the aromatic residue because it was nearly equally large for F135A and for F135K and smaller for F135Y (Fig. 4f). We speculated that the aromatic moiety of F135 stabilizes protonation of the adjacent E165 at neutral pH and that consequently D295 constitutes the exclusive counterion of the protonated retinal Schiff base as previously shown for *Ca*ChR1 from *Chlamydomonas augustae* ^39^. In line with this hypothesis, the color shift was small for the mutant E165A, but large for D295A (Fig. 4g).

For all presented mutants the high proton selectivity was preserved and reduction of extracellular protons significantly reduced photocurrent amplitude and shifted *E*_rev_ to negative values (Supplementary Fig. S6b–d).

### High proton selectivity of Chrimson is determined by the outer pore

**Figure 5:**
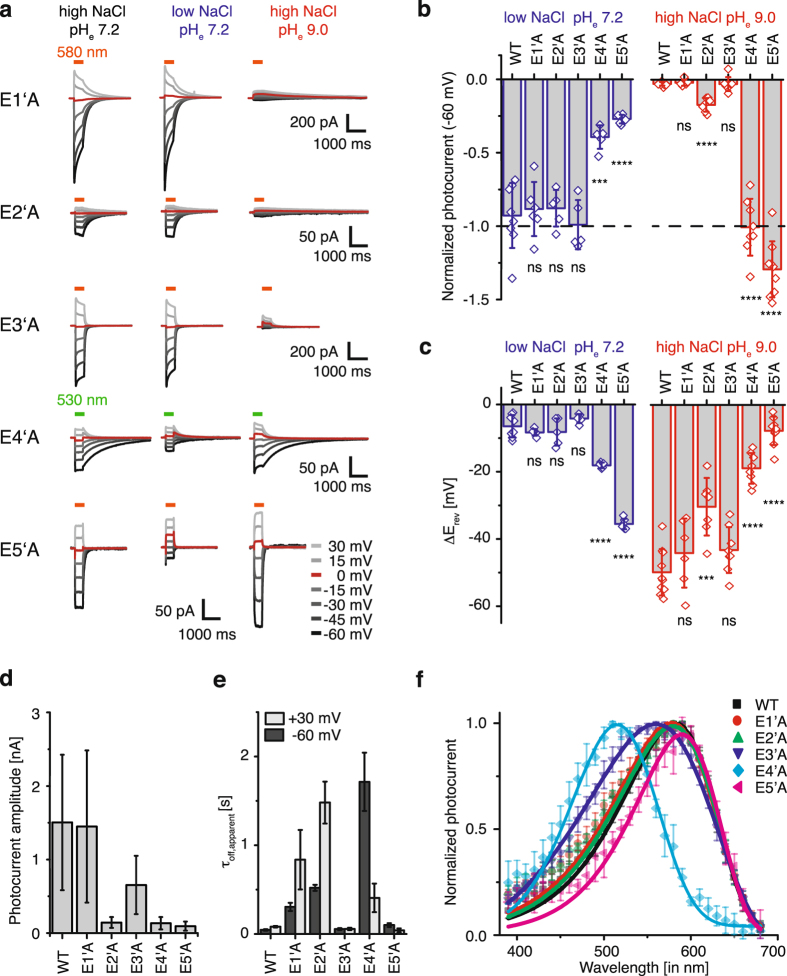
Chrimson glutamate mutants along the ion-conducting pathway. (**a**) Representative photocurrents of Chrimson glutamate mutants at different voltages and different extracellular solutions of 110 mM NaCl and pH_e_ 7.2 (left), 1 mM NaCl and pH_e_ 7.2 (middle) and 110 mM NaCl and pH_e_ 9.0 (right) with intracellular 110 mM NaCl pH_i_ 7.2. (**b**) Corresponding photocurrent amplitudes at −60 mV normalized to symmetric standard conditions of 110 mM NaCl pH_e_ 7.2 (dashed line) (Mean ± SD; LJP corrected; blue: 1 mM NaCl and pH_e_ 7.2; red: 110 mM NaCl and pH_e_ 9.0; compared to WT by a two sample t-test for “low NaCl pH_e_ 7.2”/“high NaCl pH_e_ 9.0” conditions respectively; WT: n = 8/10 cells; E1’A: n = 6/6, p = 0.7/0.4; E2’A: n = 5/7 p = 0.6/0.0002; E3’A: n = 5/8,p = 0.6/0.9; E4’A: n = 5/8, p = 0.0002/< 0.0001; E5’A: n = 6/9, p < 0.0001/0.0001) (**c**) Related reversal potential shifts upon extracellular buffer exchange from symmetric 110 mM NaCl and pH_e_ 7.2 to low extracellular sodium (left, blue, 1 mM NaCl and pH_e_ 7.2) or low extracellular proton (right, red, 110 mM NaCl and pH_e_ 9.0) concentration (mean ± SD; LJP corrected; compared to WT by a two sample t-test for “low NaCl pH_e_ 7.2”/“high NaCl pH_e_ 9.0” respectively; WT: n = 8/10 cells; E1’A: n = 6/6, p = 0.2/0.3; E2’A: n = 5/7 p = 0.45/0.0004; E3’A: n = 5/8, p = 0.11/0.06; E4’A: n = 5/8, p < 0.0001/0.0001; E5’A: n = 6/9, p < 0.0001/0.0001). (**d**) Photocurrent amplitudes at −60 mV in symmetric 110 mM NaCl and pH_i,e_ 7.2 (mean ± SD; WT n = 16 cells; E1’A n = 9; E2’A n = 10; E3’A n = 14; E4’A n = 11; and E5’A n = 10). **(e)** Apparent off-kinetics (τ_off,apparent_) at pH_e/i_ 7.2 and −60 mV or +30 mV (mean ± SD; WT n = 18 cells; E1’A n = 9; E2’A n = 8; E3’A n = 14; E4’A n = 9 cells; E5’A n = 9). (**f**) Normalized peak photocurrents after 10 ms excitation at different wavelengths of equal photon count (mean ± SD; symmetric 110 mM NaCl, pH_e,i_ 7.2 and −60 mV; WT n = 10 cells; E1’A n = 7; E2’A n = 5; E3’A = 8; E4’A n = 6; E5’A n = 5).

In *Cr*ChR2 proton and sodium conductance was modulated by site-directed mutagenesis of the highly conserved Helix2 glutamates. In particular, the central gate residue E3’ (E132 in Chrimson and E90 in *Cr*ChR2) is reportedly of high importance for sodium and proton conductance^25, 26^. To identify the specific contribution of Helix2 glutamates to proton selectivity and channel gating in Chrimson, we individually mutated the E1’ to E5’ glutamates along the putative pore to alanine residues (Fig. 5a). Confocal microscopy revealed the comparable expression of all mutants and only a modest reduction in membrane targeting for E1’A and E2’A compared to WT Chrimson (Supplementary Fig. S7 a + b). We measured photocurrents for all constructs in symmetric sodium and proton concentration and then either removed extracellular sodium (110 mM NaCl to 1 mM NaCl) or protons (pH_e_ 7.2 to pH_e_ 9.0; Fig. 5a). Unlike E3’ mutants in *Cr*ChR2, the E3’A photocurrents were not affected by the sodium reduction and removal of protons reduced the inward current (Fig. 5b) and shifted *E*_rev_ (Fig. 5c) as in the WT channel. In contrast, the E4’A and E5’A inward current amplitudes were barely affected by the removal of extracellular protons but were strongly reduced in amplitude and shifted in reversal potential after removal of extracellular sodium, indicating a major change in ion selectivity. These changes were accompanied by a reduction in the photocurrent amplitude of more than 90% (Fig. 5d). Consequently the total number of transported monovalent cations might be comparable in both mutants and the WT channel, indicating that mutation of both extracellular glutamates might not improve permeation of monovalent cations but rather impair proton conductance.

### The outer pore glutamate E4’ essentially contributes to channel gating and color shift

Interestingly, Helix2 glutamate mutation also affected gating kinetics (Fig. 5e). Whereas the photocurrent off-kinetics remained fast in E3’A and E5’A, the closing kinetics were dramatically reduced in E1’A, E2’A, and surprisingly also for E4’A, supporting their importance for channel gating. Like the adjacent Y159 in Helix 3 also E4’ in Helix2 was not only critical for channel gating but was also essential for color tuning in Chrimson (Fig. 5f). Whereas the action spectra for E1’A, E2’A, and E5’A were minimally shifted, the action spectrum of E3’A was broader and, most surprisingly, the action spectrum of E4’A exhibited a hypsochromic shift of 70 nm although E4’ is located 10 Å away from the retinal chromophore in the C1C2 structure. Interestingly, color shift of the E4’ mutant was highly related to the exact position of the carboxyl group as demonstrated by the action spectrum of the conservative mutant E4’D that showed an equilibrium of blue and orange light activation at 520 and 590 nm respectively (Supplementary Fig. S7c).

### Titratable side chains in the outer pore constitute the proton selectivity filter of Chrimson

**Figure 6:**
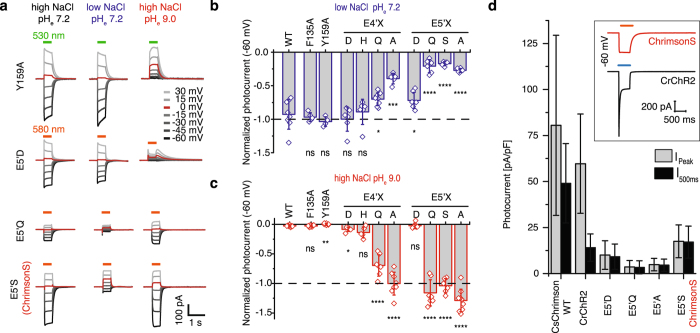
Determinants of proton selectivity in the outer pore. (**a**) Representative photocurrents of Chrimson outer pore mutants at different voltages and different extracellular solution of 110 mM NaCl and pH_e_ 7.2 (left), 1 mM NaCl and pH_e_ 7.2 (middle) and 110 mM NaCl and pH_e_ 9.0 (right) with intracellular 110 mM NaCl pH_i_ 7.2. (**b**) Photocurrent amplitudes at extracellular 1 mM NaCl pH_e_ 7.2 and −60 mV normalized to symmetric standard conditions of 110 mM NaCl pH_e_ 7.2 (dashed line) (mean ± SD; LJP corrected; compared to WT by a two sample t-test; WT: n = 8 cells; F135A: n = 5, p = 0.63; Y159A: n = 4, p = 0.24; E4’D: n = 6, p = 0.52; E4’H: n = 6, p = 0.74; E4’Q: n = 8, p = 0.025; E4’A: n = 5, p = 0.0001; E5’D: n = 7, p = 0.041; E5’Q: n = 6, p < 0.0001; E5’S: n = 6, p < 0.0001; E5’A: n = 6, p < 0.0001). (**c**) Photocurrent amplitudes at extracellular 110 mM NaCl pH_e_ 9.0 and −60 mV normalized to symmetric standard conditions of 110 mM NaCl pH_e_ 7.2 (dashed line) (mean ± SD; LJP corrected; compared to WT by a two sample t-test; WT: n = 10 cells, F135A: n = 11, p = 0.93; Y159A n = 6, p = 0.001; E4’D: n = 6, p = 0.013; E4’H: n = 5, p = 0.06; E4’Q: n = 7, p < 0.0001; E4’A: n = 8, p < 0.0001; E5’D: n = 6, p = 0.8; E5’Q: n = 7, p < 0.0001; E5’S: n = 6, p < 0.0001; E5’A: n = 9, p < 0.0001). (**d**) Peak photocurrent amplitudes (gray) and photocurrent amplitude after 500 ms illumination (black) in symmetric 110 mM NaCl and pH_i/e_ 7.2 and −60 mV normalized to the cell capacitance (mean ± SD; WT n = 16 cells; F135A n = 18; Y159A n = 6; E4’D n = 10; E4’H n = 8; E4’Q n = 8; E4’A n = 11; E5’D n = 12; E5’Q n = 8; E5’A n = 11; E5’S n = 11).

Considering the mechanism of proton selectivity in the outer pore of Chrimson we speculated that the two aromatic residues F135 and Y159 in the access channel block the passage of larger cations and might therefore – possibly held in place by E4’ and E5’ – be responsible for the high proton selectivity of Chrimson. The homolog positions have been discussed to affect ion selectivity by modulating cation binding and dehydration before^40–43^. Surprisingly, mutating both aromatic residues to the much smaller alanine and comparing photocurrent amplitudes and reversal potentials in high and low extracellular sodium and proton concentration (Fig. 6a–c) shows that neither mutation affected proton selectivity. Instead individually mutating E4’ and E5’ against titratable (E4’D, E4’H and E5’D) or non-tritratable side chains (E4’Q, E4’A, E5’Q, E5’S, E5’A) shows that high proton selectivity is significantly impaired by unprotonatable side chains at the position of E4’ and especially E5’ (Fig. 6a–c). Again, reduction of proton selectivity comes along with a strong reduction in photocurrent amplitude (Fig. 6d). However under prolonged illumination, photocurrents of E5’S neither decline as WT photocurrents nor inactivate as photocurrents of *Cr*ChR2 and even surpass the late photocurrent amplitude of *Cr*ChR2 after 500 ms activation (Fig. 6d Inlet). Red light activation, increased sodium selectivity (P_H+_/P_Na+_ = 5.3 ± 0.2 *10^5^) with photocurrents carried to more than 80% by sodium at pH_e,i_ 7.2 and stable photocurrent amplitude at continuous illumination uniquely classify Chrimson E5’S, which we term ChrimsonS(odium), as an highly promising optogenetic tool for prolonged red light depolarization of excitable cells.

## Discussion

Despite the established conductance of ChRs for different mono- and divalent ions (Li > Na^+^ > K^+^ > Cs^+^ ≫ Ca^2+^ > Mg^2+^)^14, 37^ ChRs are first of all highly selective for protons (P_H+_/P_Na+_ = 2–6 × 10^6^ in *Cr*ChR2)^14, 44, 45^. The proton selectivity of ChRs is much higher than that of other cation channels such as ASIC1a (P_H+_/P_Na+_ ≥ 5^46^), Gramicidin A (P_H+_/P_Na+_ = 43–55^47^) or TRPV1 (P_H+_/P_Cs+_ = 87-1127^48^) and is in the same range with highly selective proton channels including the influenza virus M2 channel (P_H+_/P_Na+_ = 10^5^-10^7^ ^49–51^) or the voltage-gated proton channel H^+^H_v_1 (P_H+_/P_Na+_ ≥ 10^6^ ^52–54^). Although the ChR-conducting pore was localized between helices 1, 2, 3, and 7 ^19, 20, 23^ the precise contribution of the pore flanking side chains is still unclear due to the lack of an open pore structure.

In the present work we analyzed channel gating and ion selectivity of the red light activated channelrhodopsin Chrimson that due to the unique opsin shift to 585 nm and the large photocurrents at neutral pH represents one of the most promising optogenetic actuators. We showed that Chrimson features a distinct ion selectivity compared to other channelrhodopsins with reduced permeability for divalent ions such as Ca^2+^ and Mg^2+^ but significantly increased proton selectivity. In contrast to other proton selective channelrhodopsins such as *Ds*ChR1 from *Dunaliella salina* ^4^ proton conductance of Chrimson is also high at neutral pH indicating an improved proton permeation pathway.

During prolonged illumination Chrimson features a slow photocurrent decline that could easily be misinterpreted as slow channel inactivation. However by correlating it with channel expression, proton buffer concentration and intracellular acidification, we show that it does not represent an inactivation of the ion channel itself, but rather a decline in the electrochemical driving force due to changes in the pH gradient. Considering that re-equilibration of the intracellular pH through the patch pipette is slow (5 s to several min)^55–57^ due to the geometrical constraints of the small pipette tip and the slow intracellular proton diffusion^58–60^, it can be assumed that the low intracellular proton concentration is perturbed by the large proton currents of Chrimson. Moreover, similar intracellular pH changes due to large proton fluxes were reported and discussed for voltage-gated proton channels before^61, 62^. Interestingly slow photocurrent decline during prolonged illumination has been reported also for other ChRs such as *Ca*ChR1, *Cy*ChR1 or Chronos^29, 63^ Consequently intracellular acidification might be a general side effect of prolonged activation of proton conductive ChRs, that - depending on the relative proton permeability - might result in varying degrees of photocurrent reduction.

**Figure 7:**
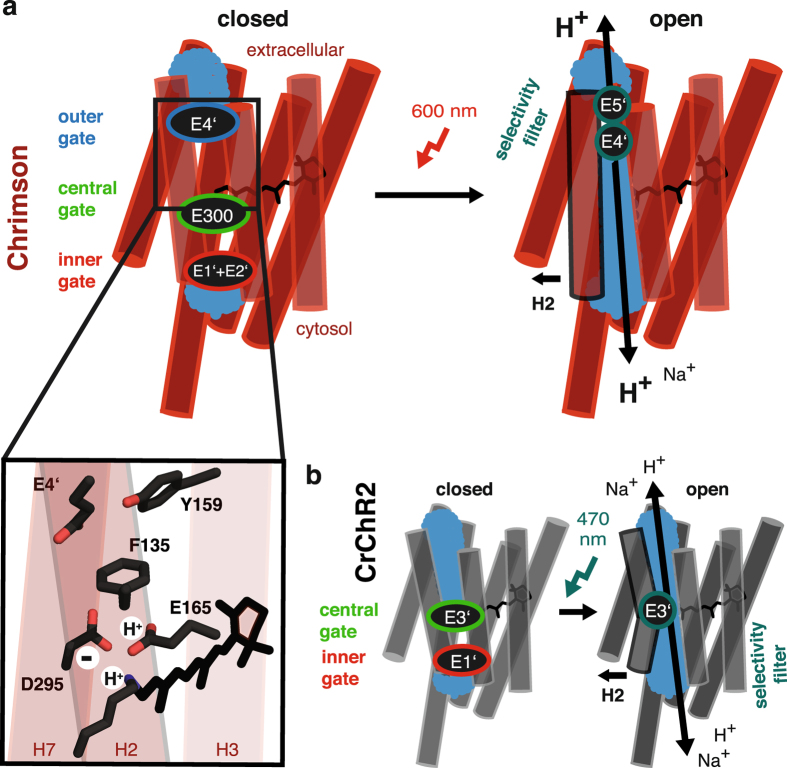
Gate structure, open pore conformation and counterion configuration in Chrimson. (**a**) Schematic putative closed and open configuration of Chrimson. Closed: Non-conductive conformation of Chrimson that is stabilized by E1’ and E2’ in the inner gate, E300 in the central gate and E4’ in the outer gate. Open: Conductive conformation of Chrimson with a pore constriction and proton selectivity filter in the outer pore at E4’ and E5’ Inlet: Counterion configuration with a protonated retinal Schiff’base, protonated E165 and deprotonated D295. Mutants of all presented residues induce significant hypsochromic shifts, indicating an important role of displayed side chains in counterion configuration (F135A Δλ_max_ = 50 ± 6 nm, E4’A Δλ_max_ = 70 ± 5 nm, Y159A Δλ_max_ = 39 ± 4 nm, E165A Δλ_max_ = 20 ± 5 nm, D295A Δλ_max_ = 61 ± 7 nm). (**b**) Comparison to the proposed configurations in *Cr*ChR2. Closed: Non-conductive conformation of *Cr*ChR2 that is stabilized by E1’ and R310 in the inner gate and E3’, S63 and N258 in the central gate. Open: Conductive state of *Cr*ChR2 with a pore constriction and proton selectivity filter in the center of the protein at E3’.

High proton selectivity of Chrimson is essentially determined by titratable outer pore residues indicating a different open pore confirmation compared to previously characterized ChRs (Fig. 7a right). In *Cr*ChR2 ion conduction is initiated by subsequent water influxes and pore hydration^22^. Nevertheless, a continuous water-filled pore alone, as in the highly proton conductive but poorly selective Gramicidin A channel^64, 65^, would not account for the high proton selectivity. However, transmembrane proton transport does not require a continuous water filled pore but may proceed along hydrogen bonding or protonatable amino acid side chains^66^, passing protons along the wire while occluding the passage of other ions. In the voltage gated proton channel H^+^Hv1, a single titratable aspartate (D112) located at a putative pore constriction is essential for proton selectivity, and the mutation of the same residue converted H^+^Hv1 to a chloride channel^67^. We confirm for Chrimson that also in ChRs single titratable residues are crucial for proton selectivity. The observation that mutation of central gate E3’ to a neutral side chain strongly reduces proton conductance for *Cr*ChR2^26^ but had no effect in Chrimson, whereas mutating E4’ and E5’ in the outer pore decreased proton selectivity in Chrimson but not in *Cr*ChR2^25, 40^ reveals that the proton selectivity filter can be located at different position along the conductive pore and implies different open pore constrictions at the outer pore for Chrimson and in the center of the protein for *Cr*ChR2 (Fig. 7a + b). An additional and distinct open pore constriction in Chrimson is further supported by the observation of the proton and sodium permeation block by the organic cation guanidinium that - although the best conducted ion in *Cr*ChR2^14^ - permeates Chrimson only at a negligible rate. A recent theoretical study concluded on an Asp-Arg-pair as the proton selectivity filter in H^+^Hv1^68^ preventing anion^67^, cation and especially guanidinium permeation^69^. Interestingly, E4’ and E5’ are located in close proximity of the highly conserved R162 (Fig. 4a), which serves in bacteriorhodopsin and relatives as a proton shuttle changing orientation and interacting with extracellular glutamates during proton pumping. Thus, it is tempting to speculate that also in Chrimson a Glu-Arg pair might contribute to proton selectivity, although less rigid considering the residual cation conductance. However, further theoretical and ideally structural studies are required to resolve the molecular nature – eventually including ordered waters - of the Chrimson selectivity filter. Because mutation in the inner half channel does not affect proton selectivity although affecting overall photocurrent amplitude, we assume wider open pore dimensions in the inner half channel of Chrimson and conformational rearrangements including outward movement of Helix2 and subsequent water influx as observed for *Cr*ChR2^19, 20, 23, 70^ might be conserved.

Channel gating of Chrimson is controlled by four pore lining glutamates – namely E1’(E124) and E2’(E125) in the inner gate, E300 in the central gate and E4’(E139) in the outer pore (Fig. 7a left). Mutation of each of these glutamates severely retarded channel closure, whereas in *Cr*ChR2 only mutation of E1’ significantly decelerated closing kinetics^40, 71^. We conclude that Chrimson features an altered gate structure with major rearrangements in the inner gate and central gate and an additional outer gate organized around E4’ that occludes the outer pore in the dark and contributes to the proton selectivity filter during illumination – as discussed above. The four pore lining glutamates could assist channel closure by stabilizing the closed pore through interhelical hydrogen bonds or electrostatic interactions as proposed for E1’-R310 and E2’-H134 in the inner gate and E3’- N257 in the central gate of *Cr*ChR2. Although it is difficult to identify hydrogen bonding partners of the four Chrimson glutamates without a high resolution crystal structure, we can exclude electrostatic interaction of E2’ with the H134 homolog K176 as well as interhelical hydrogen bonding of the N257 homolog E300 with E3’, because neither K176 nor E3’ decelerated closing kinetics - further supporting different inner and central gate arrangements in Chrimson. On the contrary channel closure is even accelerated in the mutant K176A and unlike in WT independent of extracellular pH_e_, indicating that K176 even disfavors pore closure in the inner gate, which follows transitions in the extracellular half pore in WT but becomes rate limiting in the mutant. Alternatively, pore lining glutamates could also contribute to the process of channel closing by facilitating proton transfer reactions. Accordingly, channel closure of E300A is - although still slower than WT - 20 times faster than in E300N. Although E300A can not contribute to interhelical interaction it allows a water molecule to replace the E300 acid residue, supporting that E300 due to its location close to the retinal counter ion and the retinal Schiff’base, contributes to proton transfer reactions during channel closing. The observation that mutants of E2’ are slow at positive voltages whereas E4’ mutants are slow at negative voltage further indicates that different proton transfer reactions from the inside or the outside of the cell are rate limiting at different voltages. Engagement of protons of the conducting pathway into channel *off*-gating is not unlikely, because essential amino acid that contribute to proton transfer reactions in *Cr*ChR2 such as D156^72^ are not conserved.

Mutation of the outer pore residues E4’(E139) and the adjacent Y159 caused large hypsochromic shifts of 70 nm and 40 nm respectively in spectral sensitivity that are surprising due to the distance of the mutated residue to the retinal chromophore in the C1C2 structure (Fig. 7a inlet). The opsin shift in rhodopsins is essentially determined by the planarity of the conjugated system of the retinal, the polarity and charges of residues at the ß-ion ring and by electrostatic interaction of the counter ion and the protonated retinal Schiff base^73^. Whereas in *Cr*ChR2 both carboxylic acids of the counterion complex are negatively charged^74^, our action spectra indicate that in Chrimson D295 constitutes the exclusive counterion of the protonated Schiff’base and E165 is protonated at neutral pH. Protonation of E165 is crucial for red light absorption and might be essentially stabilized by F135 - similar as in *Ca*ChR1^39^ – but also by the outer gate residue E4’ and the adjacent Y159. Although we can exclude direct interaction of E4’ and Y159 with the counterion complex due to their relative distances, both residues could influence protonation and geometry of the counter ion complex via an unidentified third residue, a structured water or by interhelical hydrogen bonding involved in counterion positioning. Whereas a narrow outer pore conformation – possibly featuring additional structured water – is supported by our selectivity analysis, slow photocurrent kinetics of E4’ mutants also suggest alternative interhelical hydrogen bonding in the outer gate. The bifurcated activation of E4’D support both interaction schemes and underlines the sophisticated engagement of outer pore residues into counter ion confirmation that is unique in Chrimson. Interestingly peak activity of the F135K in Chrimson at 520 nm is still 40 nm red shifted compared to the homolog mutation in *Ca*ChR1^39^ confirming that in addition to the counterion configuration also structural differences in the retinal binding pocket essentially contribute to the large opsin shift of Chrimson.

With the presented experiments we functionally characterized ion permeation of the red-light activated ChR Chrimson. Whereas the lack of calcium permeation is beneficial for optogenetic applications, in experiments involving extended or high frequency illumination the high proton selectivity of Chrimson could cause side effects and unstable light response due to intracellular acidification and subsequent slow photocurrent decline especially in small cellular compartments such as synapses or dendrites. Identifying key determinants of channel gating, proton selectivity and red-light activation we localized an additional activation gate in the outer pore that – not found in other ChRs - is essential for fast channel closure, high proton conductance and red light activation of Chrimson. We generated Chrimson variants with significantly reduced or elongated open state lifetimes (K176A and E300N) or highly improved sodium selectivity (ChrimsonS) that preserve red light activation and substantially complement the optogenetic toolbox of red light activated actuators. Similarities in the selectivity mechanism in Chrimson and *Cr*ChR2 – imposed by individual titratable residues - and differences in the localization of the selectivity filter and channel gates – inner, central and outer gate - constitute an essential complementation of our understanding of ChRs and contribute to the transition from a *Cr*ChR2 centric perception to a more general understanding of the ChR protein family.

## Methods

### Cloning, site-directed mutagenesis and preparation of HEK-293 cells

In the present work three different targeting variants of Chrimson were employed. The coding sequences of Chrimson (KF992060.1) and CsChrimson containing the extracellular N-terminus from *Cs*ChR of *Chloromonas subdivisa* (KJ995863.2, in a FCK-GFP vector) were provided by Ed Boyden (MIT, Boston, MA) and cloned into the pmCerulean-C1 vector using Nhe1 and Age1 restriction sites. For the design of ßHK_Chrimson, the first 105 N-terminal amino acids of the rat gastric H+/K+ -ATPase beta subunit (NM_012510.2) were fused to the N-terminus of Chrimson and cloned into a peCFP-C1 vector for electrophysiological recordings and pH-imaging, into a pmCherry-N1 vector for calcium imaging, or into pmCerulean3.0-C1 for confocal imaging. Chrimson expression and photocurrents were nearly unaffected by the targeting strategy (Supplement Fig. S1). We used ßHK_Chrimson for the basic characterization of Chrimson photocurrents (Figs 1–3 + Supplementary Figs S2–S4) and CsChrimson as a backbone for site-directed mutagenesis (Figs 4–6 + Supplementary Figs S6–S7) (both constructs are abbreviated as “Chrimson” throughout the manuscript). Site-directed mutagenesis was performed using the QuickChange Site-Directed Mutagenesis Kit (Agilent Technologies, Santa Clara, CA) according to the manufacturers’ instructions.

For comparison of Chrimson proton selectivity the coding sequences of C1C2 (3UG9_A) and *Ps*ChR (AGF84747.1; Genescript, Piscataway, NJ) were cloned into the pmCherry-N1 vector using the HindIII and BamHI or NheI and AgeI restriction sites respectively.

All constructs were expressed under the control of a CMV-promotor. HEK293 cells were cultured in Dulbecco’s Modified Medium (DMEM) with stable glutamine (Biochrom, Berlin, Germany) supplemented with 10% (v/v) fetal bovine serum (FBS Superior; Biochrom, Berlin, Germany), 1 μM all-*trans* retinal, and 100 µg/ml penicillin/streptomycin (Biochrom, Berlin, Germany). The cells were seeded on coverslips at a concentration of 0.75 × 10^5^ cells/ml and transiently transfected using the FuGENE® HD Transfection Reagent (Promega, Madison, WI) 28–48 h before measurement.

### Patch-clamp experiments in HEK293 cells

Patch pipettes were prepared from borosilicate glass capillaries (G150F-3; Warner Instruments, Hamden, CT) using a P-1000 micropipette puller (Sutter Instruments, Novato, CA) and were subsequently fire polished. The pipette resistance was between 1.8 and 3.0 MΩ. Single fluorescent cells were identified using an Axiovert 100 inverted microscope (Carl Zeiss, Jena, Germany). Monochromatic light (±7 nm) was provided by a Polychrome V monochromator (TILL Photonics, Planegg, Germany), attenuated either by a motorized neutral density filter wheel (Newport, Irvine, CA) for equal photon fluxes at different excitation wavelengths, or by different neutral density filters (Schott, Mainz, Germany) for light titration experiments and was temporally controlled by a VS25 and VCM-D1 shutter system (Vincent Associates, Rochester, NY). Recorded signals were filtered at 2 kHz using an AxoPatch 200B amplifier (Molecular Devices, Sunnyvale, CA) and digitized using a DigiData 1440 A digitizer (Molecular Devices, Sunnyvale, CA) at a sampling rate of 5–10 kHz. The reference bath electrode was connected to the bath solution via a 140 mM NaCl agar bridge. The extracellular buffer exchange was performed manually by adding at least 5 ml of the respective buffer to the recording chamber (500 µl chamber volume) while a Ringer Bath Handler MPCU (Lorenz Messgerätebau, Katlenburg-Lindau, Germany) maintained a constant bath level. Standard bath solutions contained 110 mM NaCl, 1 mM KCl, 1 mM CsCl, 2 mM CaCl_2_, 2 mM MgCl_2_ and 10 mM HEPES at pH_e_ 7.2 (with glucose added up to 310 mOsm). Standard pipette solution contained 110 mM NaCl, 1 mM KCl, 1 mM CsCl, 2 mM CaCl_2_, 2 mM MgCl_2_, 10 mM EGTA and 10 mM HEPES at pH_i_ 7.2 or 10 mM TRIS at pH_i_ 9.0 (glucose added up to 290 mOsm). For ion selectivity measurements NaCl was replaced by either 110 mM KCl, 110 GuanidiniumCl, 110 mM NMDGCl, 55 mM CaCl_2_ or 55 mM MgCl_2_ with 1 mM NaCl remaining. Solutions at pH_e_ 9.0 were buffered with 10 mM TRIS and solutions at pH_e_ 5.0 with 10 mM citrate buffer instead of HEPES. The light intensities were measured after passing through all of the optics using a P9710 optometer (Gigahertz-Optik, Türkenfeld, Germany) and normalized to the water Plan-Apochromat 40×/1.0 differential interference contrast (DIC) objective illuminated field (0.066 mm^2^). The maximum light intensity was 2.37 mW × mm^−2^ at 600 nm, 2.28 mW × mm^−2^ at 580 nm and 2.47 mW × mm^−2^ at 530 nm. All electrical recordings were controlled by the pCLAMP™ software (Molecular Devices, Sunnyvale, CA). The whole-cell recordings had a minimum membrane resistance of 500 MΩ (usual > 1 GΩ) and an access resistance below 10 MΩ.

### pH Imaging with BCECF

#### Intracellular calibration of BCECF

HEK293 cells were visualized on an Olympus iX70 inverted microscope (Olympus, Tokyo, Japan) and incubated with a loading solution composed of Dulbecco’s phosphate-buffered saline (DPBS), 2 µM BCECF-AM (Molecular Probes), 250 µM sulfinpyrazone, and 0.004% (m/V) Pluronic® F-127 (Molecular Probes) for 5 min at room temperature. After removing the loading solution, the cells were incubated for an additional 5 min in DPBS for AM ester hydrolyses. An *in situ* pH calibration was performed by adding 20 µM nigericin, a K^+^/H^+^ ionophore (Molecular Probes), to calibration buffers containing: 135 mM KCl, 2 mM K_2_HPO_4_, 1.2 mM CaCl_2_, 0.8 mM MgSO_4_, and 20 mM HEPES at pH_e_ 6.0, 6.5, 7.0, 7.2, 7.5, and 8.0 (glucose added up to 320 mOsm). Monochromatic excitation light (±7 nm) was supplied by a Polychrome V monochromator (TILL Photonics, Planegg, Germany) and directed through a Fluar 40×/1.30 oil objective (Carl Zeiss Microscopy, Thornwood, NY) using the 444/520/590 HC triple band dichroic mirror and the 465/537/623 HC triple band emission filter (AHF Analystechnik, Tübingen, Germany). Imaging experiments were controlled by the TILLvisION software (TILL Photonics, Planegg, Germany). After excitation at 500 nm, BCECF fluorescence was probed every 45 s with an exposure time of 5 ms and an intensity of 0.4 mW × mm^−2^ and was measured using a CCD Imago camera (TILL Photonics, Planegg, Germany). The calibration solutions were added manually to the measuring chamber in a varying sequence and were removed using a peristaltic pump (Watson-Marlow, Falmouth, UK) at moderate speed.

#### pH imaging during patch-clamp recordings

For simultaneous BCECF imaging and voltage-clamp measurements, 100 µM BCECF acid (Molecular Probes) was added to the pipette solution and dispersed into the cytoplasm after establishing the whole-cell configuration. Electrical recordings were performed as previously described and were controlled and synchronized to TILLvisION imaging software using pCLAMP®. ChR activation was achieved using a 75 W Xenon lamp (Jena-Instruments, Jena, Germany), the light duration was controlled using a VS25 and VCM-D1 shutter system (Vincent Associates, Rochester, NY), and the wavelength was selected by a band pass filter centered at 560 nm with a full width at half maximum (FWHM) of 20 nm. Activation light at 560 nm was combined with the beam from the polychrome unit via a 70 R/30 T beam splitter. During the electrical recordings, the ChRs were activated at 560 nm for 15 s at different voltages at a light intensity of 0.8 mW × mm^−2^ and BCECF fluorescence after excitation at 500 nm was recorded simultaneously at an exposure time of 5 ms and a sampling rate of 2 Hz. The electrical recordings during pH imaging had a membrane resistance higher than 300 MΩ (typical ~1 GΩ) and an access resistance below 15 MΩ (typical < 10 MΩ).

### Calcium imaging with Fura-2

For calcium imaging, HEK293 cells were incubated for 5 min in DMEM (Biochrom, Berlin, Germany) supplemented with 2 µM Fura-2-AM (Molecular Probes) before transferring the sample to the Olympus iX70 inverted microscope (Olympus, Tokyo, Japan). Using the same experimental setup used for the pH imaging experiments, ChRs were activated using the 75W-Xenon lamp and band pass filters centered at 450 nm (FWHM = 55 nm) for the activation of *Cr*ChR2 T159C and at 560 nm (FWHM = 20 nm) for the activation of Chrimson. For ratiometric measurements, Fura-2 was excited at 340 nm and 380 nm using the Polychrom V unit. Both illumination paths were combined with a 70 R/30 T beam splitter and guided via a FF493/574 dichroic mirror (AF-Analysetechnik, Tübingen, Germany) to the Fluar 40×/1.30 oil objective. For sufficient light intensities at 340 nm, UV-transparent, fluorescent-free Fluka immersion oil (Sigma-Aldrich) was used. The resulting light intensities were 2.9 mW/mm^2^ for the activation of *Cr*ChR2 T159C at 450 nm, 0.9 mW/mm^2^ for the activation of Chrimson at 560 nm, and 0.004 mW/mm^2^ and 0.1 mW/mm^2^ for the excitation of Fura-2 at 340 nm and 380 nm, respectively. The fluorescence of Fura-2 was recorded at exposure times of 200 ms and 25 ms at 340 nm and 380 nm, respectively, and at a sampling rate of 0.5 Hz. The ChRs were excited in the corresponding maximum of their action spectrum for 10 s. For the calcium imaging experiments, the region of interest for Fura-2 imaging was searched using a standard extracellular buffer containing low extracellular calcium (2 mM) before switching to higher calcium concentrations for Fura-2 imaging. The ion composition during the recordings was 70 mM CaCl_2_, 2 mM MgCl_2_, 1 mM KCl, 1 mM CsCl, 1 mM NaCl, and 10 mM HEPES at pH_e_ 7.2 and glucose up to 310 mOsm.

### Confocal microscopy

The subcellular localization of the Chrimson constructs in HEK293 cells was monitored 35 h after transfection using a FluoView™ 1000 confocal microscope system (Olympus, Tokyo, Japan). The cell membrane was labeled with 2 μM octadecyl rhodamine B chloride (R18; Molecular Probes), and pictures were acquired on a confocal LSM IX81 microscope equipped with a 60 × 1.2 water-immersion UplanSApo objective (Olympus, Tokyo, Japan). A 440 nm laser diode operating at 10–14% and a 559 nm laser diode operating at 2–5% were used to excite mCerulean3.0 and R18, respectively. The fluorescence emission was sequentially detected at 476 nm and 591 nm, respectively, using a photomultiplier tube.

### Data analysis

The analysis of the electrical recordings was performed using Clampfit 10.4 software (Molecular Devices, Sunnyvale, CA) and the imaging data were analyzed with the TILLvisION software. Statistical analyses were performed in Microsoft Excel and Origin 9.1® (OriginLab, Northampton, MA). Photocurrent traces were baseline corrected, filtered, and reduced in size for display purposes. Photocurrents were normalized to peak photocurrents at −60 mV under standard conditions of extracellular 110 mM NaCl pH_e_ 7.2. The liquid junction potentials (LJP) were calculated in Clampex 10.4 and the applied voltages were corrected offline if stated so in the figure legend. The reversal potentials were estimated by a linear interpolation of the two data points adjacent to the photocurrent direction inversion. For comparison of photocurrent amplitudes at different LJP, the photocurrent amplitudes of the two adjacent data points were linearly extrapolated to the designated voltage. Action spectra were fitted using a parametric Weibull function (y = y0 + A*((w2 − 1)/w2)^((1 − w2)/w2)*S^(w2 − 1)*exp(−s^w2 + (w2 − 1)/w2) with S = (x − xc)/w1 + ((w2 − 1)/w2)^(1/w2) and the estimated parameters A, y0, w1, w2 and xc). The photocurrent kinetics were estimated by mono- or biexponential fits and simplified by an apparent time constant (τ_apparent_) calculated as (A_1_*τ_1_ + A_2_* τ _2_)/(A_1_ + A_2_). The confocal images for the evaluation of membrane targeting were analyzed in FluoView FV10-ASW 3.0 (Olympus Europa, Hamburg, Germany) and using the Fiji image processing package^75^. The membrane region was localized by the R18 fluorescence and the mean mCerulean3.0 fluorescence in the membrane region was quantified and compared with the mean intracellular fluorescence. For sufficient statistical significance each measurement was repeated multiple times on different biological replicates in at least two independent experiments. The exact number of biological replicates for each measurement is provided in the figure legend. To compare the data, we performed two-sample t-tests with Welch’s correction in Origin 9.1®. The significance thresholds were set at p < 0.05 (*), p < 0.01 (**), p < 0.001 (***), and p < 0.0001 (****).

## Electronic supplementary material

Supplementary information
