# Supplementary material for: Molecular determinants of proton selectivity and gating in the red-light activated channelrhodopsin Chrimson
Source: Sci Rep. 2017 Aug 30;7:9928. doi: 10.1038/s41598-017-09600-8 (PMC5577340; doi:10.1038/s41598-017-09600-8)
Supplement: Supplementary file 1 — Supplementary information [file 41598_2017_9600_MOESM1_ESM.pdf]

Molecular determinants of proton selectivity and gating in the red-light activated channelrhodopsin Chrimson

Supplementary Figures S1-S7

Johannes Vierock*, Christiane Grimm, Noam Nitzan, Peter Hegemann

Institute of Biology, Experimental Biophysics, Humboldt-Universität zu Berlin,

10115 Berlin, Germany

* To whom correspondence may be addressed: Johannes Vierock, Experimental Biophysics, Institute of Biology, Humboldt-Universität zu Berlin, Invalidenstr. 42, 10115 Berlin, Germany, Tel.: +49 30 2093-8897; Fax: +49 30 2093-8520; E-mail: vierockj@biologie.hu-berlin.de

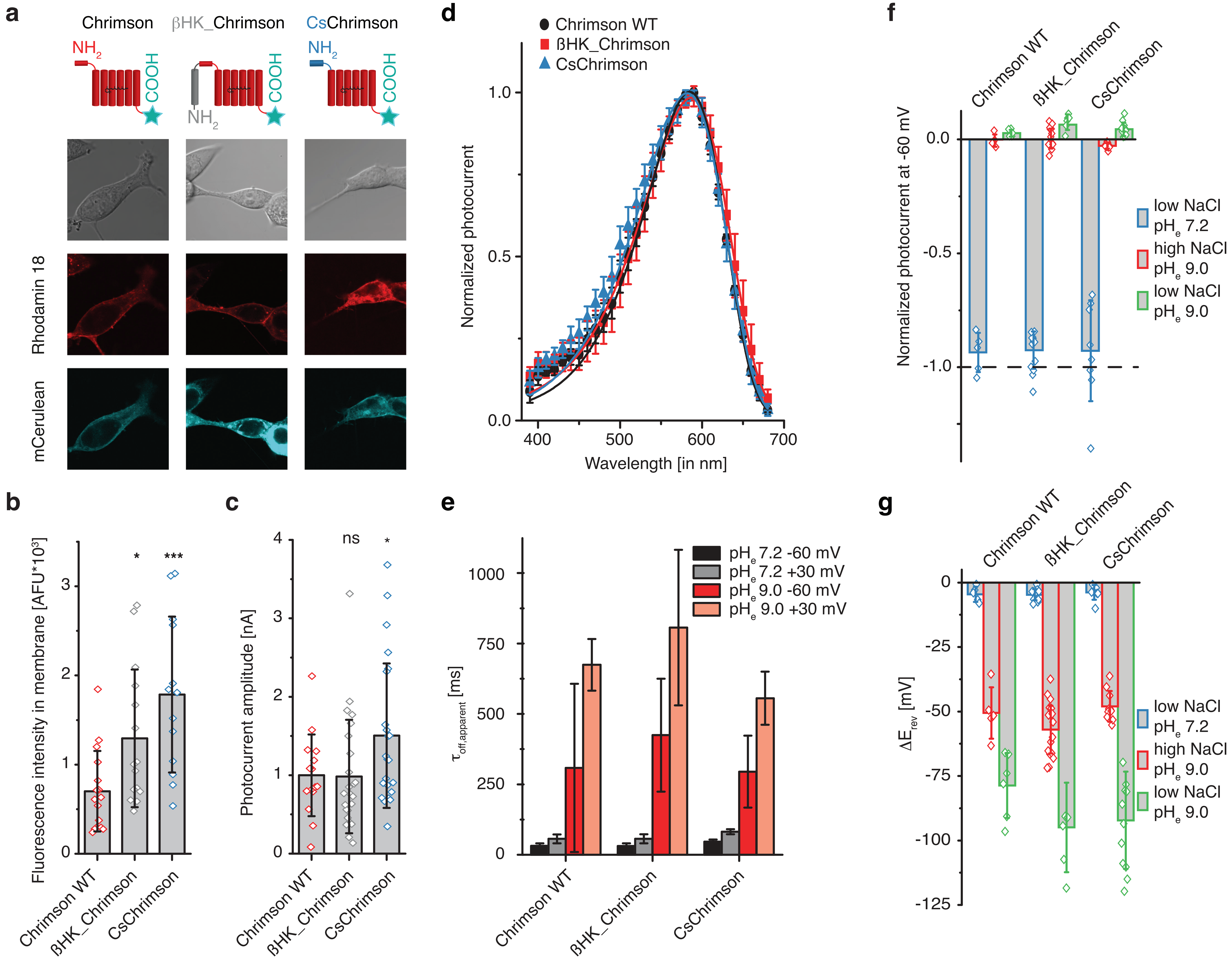

Supplementary Figure S1: Head-to-head comparison of Chrimson targeting constructs (a) Top: Protein schemes of targeting variants with WT Chrimson (red), the H^+/K^+ ATPase βHK-subunit (gray), the N-terminus of CsChR (blue) and C-terminal mCerulean3.0 (cyan). Below: Confocal images of HEK293 cells illuminated by transmitted light, using 559 nm for the excitation of Rhodamin18 and 440 nm for mCerulean3.0. **(b)** mCerulean3.0 fluorescence intensity in the membrane region (mean ± SD, compared to Chrimson WT by a two sample t-test; Chrimson WT: n=16 cells; βHK_Chrimson: n=15, p=0.017; CsChrimson: n=13, p=0.0008). **(c)** Photocurrent amplitudes at -60 mV in symmetric 110 mM NaCl and $pH_{e,i}$ 7.2 (mean ± SD; compared to Chrimson WT by a two sample t-test; Chrimson WT: n=15 cells; βHK_Chrimson n=24, p=0.94; CsChrimson: n=23, p=0.037). **(d)** Normalized peak photocurrents after 10 ms excitation at different wavelengths of equal photon count (mean ± SD; symmetric 110 mM NaCl, $pH_{e,i}$ 7.2 and -60mV; Chrimson WT n=6 cells, βHK_Chrimson n=9, CsChrimson n=10). **(e)** Apparent off-kinetics ($\tau_{apparent,\ off}$) at positive and negative voltages and pH_e 7.2 and pH_e 9.0 (mean ± SD; Chrimson WT n=7–15 cells; βHK_Chrimson n=15–36; CsChrimson n=12–18). **(f)** Photocurrent amplitudes at -60 mV and low extracellular sodium (left, blue, 1 mM NaCl pH_e 7.2), low extracellular proton (middle, red, 110 mM NaCl pH_e 9.0) and low extracellular sodium and proton concentrations (right, green, 1 mM NaCl pH_e 9.0) normalized to symmetric standard conditions of 110 mM NaCl pH_e 7.2 (dashed line) (Mean ± SD; LJP corrected; Chrimson WT: n=5-6 cells; βHK_Chrimson: n=6–16; CsChrimson: n=8–12). **(g)** Reversal potential shifts upon extracellular buffer exchange from symmetric 110 mM NaCl and pH_e 7.2 to low extracellular sodium (left, blue, 1 mM NaCl and pH_e 7.2), low extracellular proton (middle, red, 110 mM NaCl and pH_e 9.0) or low extracellular sodium and proton (right, green, 1 mM NaCl pH_e 9.0) concentration (mean ± SD; LJP corrected; Chrimson WT: n=5-6 cells; βHK_Chrimson: n=6–16; CsChrimson: n=8–12).

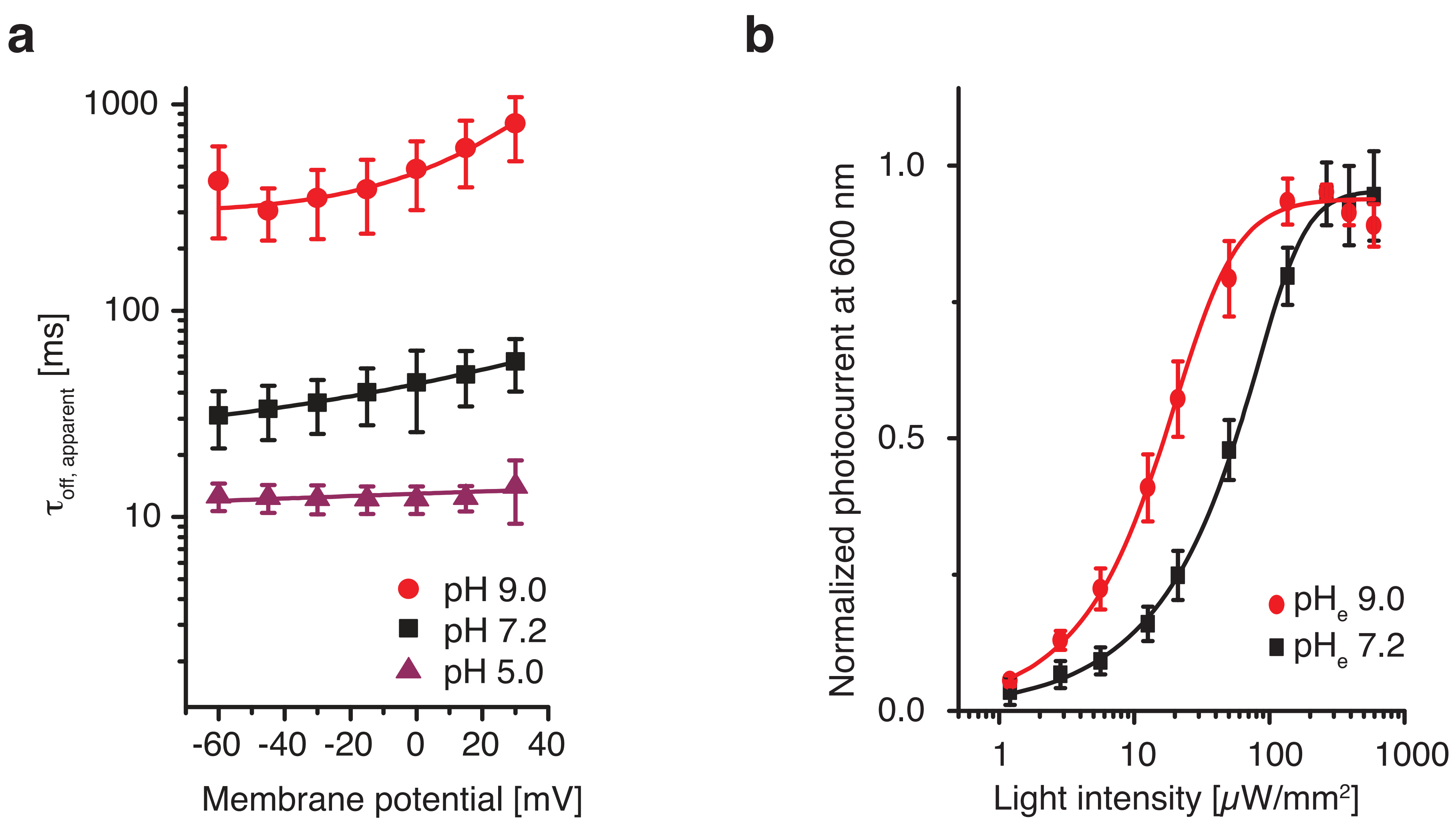

Supplementary Figure S2: Channel closure kinetics and light sensitivity at different extracellular pH_e (a) Voltage dependence of apparent off-kinetics of Chrimson in symmetric 110 mM NaCl and different extracellular pH_e(mean ± SD; n=8-36 cells). **(b)** Photocurrents in dependence of the intensity of the actinic light (500 ms illumination) at pH_e 7.2 and 9.0 normalized to maximal response (mean ± SD; 110 mM NaCl and -60mV; n=6-12 cells).

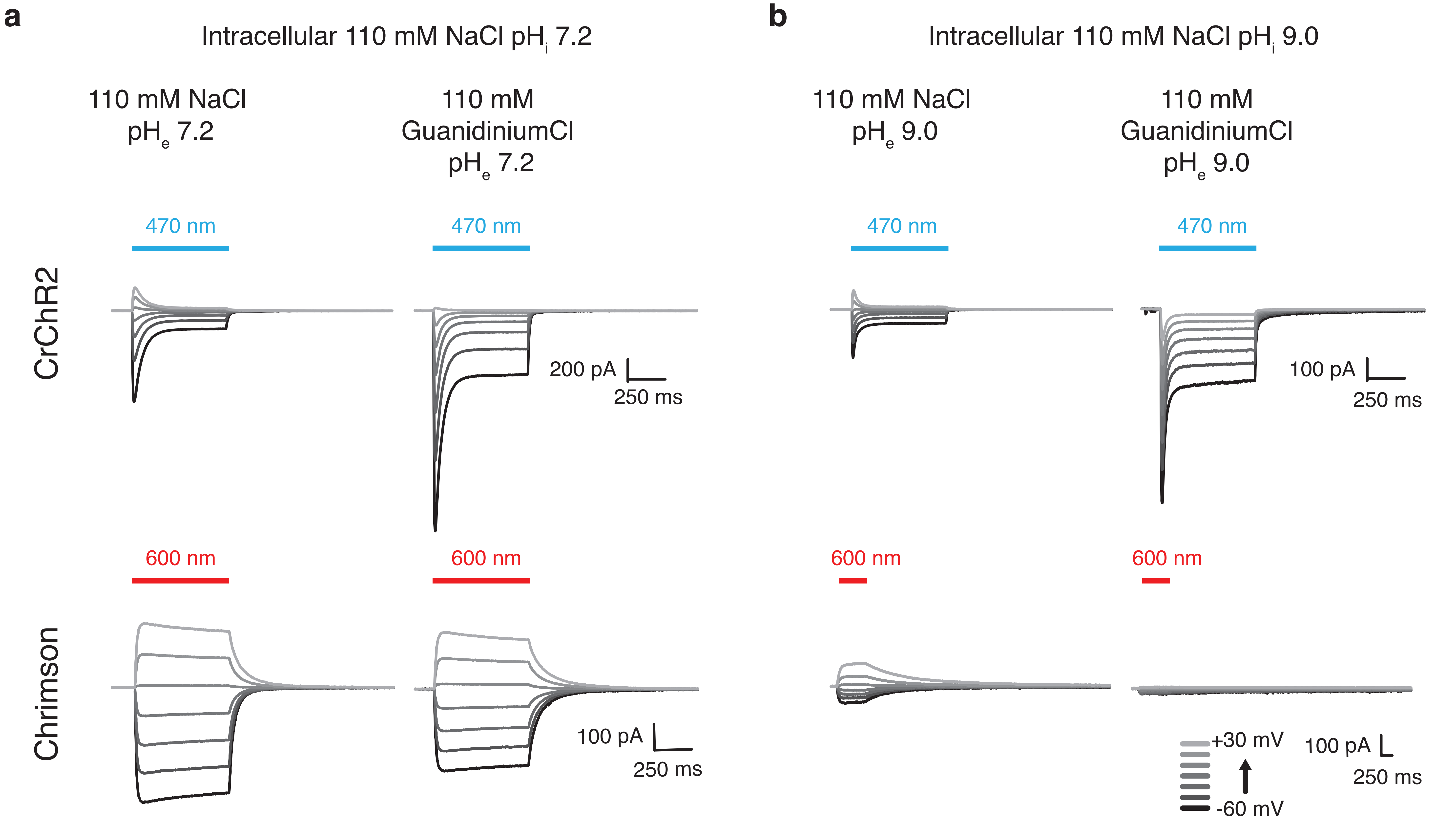

Supplementary Figure S3: Guanidinium conductance of CrChR2 and Chrimson (a) Representative photocurrents of CrChR2 (top) and Chrimson (bottom) at different voltages and extracellular 110 mM NaCl and pH_e 7.2 (left) and 110 mM GdmCl pH_e 7.2 (right) with intracellular 110 mM NaCl pH_i 7.2. **(b)** Representative photocurrents of CrChR2 (top) and Chrimson (bottom) at different voltages and extracellular 110 mM NaCl and pH_e 9.0 (left) and 110 mM GdmCl pH_e 9.0 (right) with intracellular 110 mM NaCl pH_i 9.0.

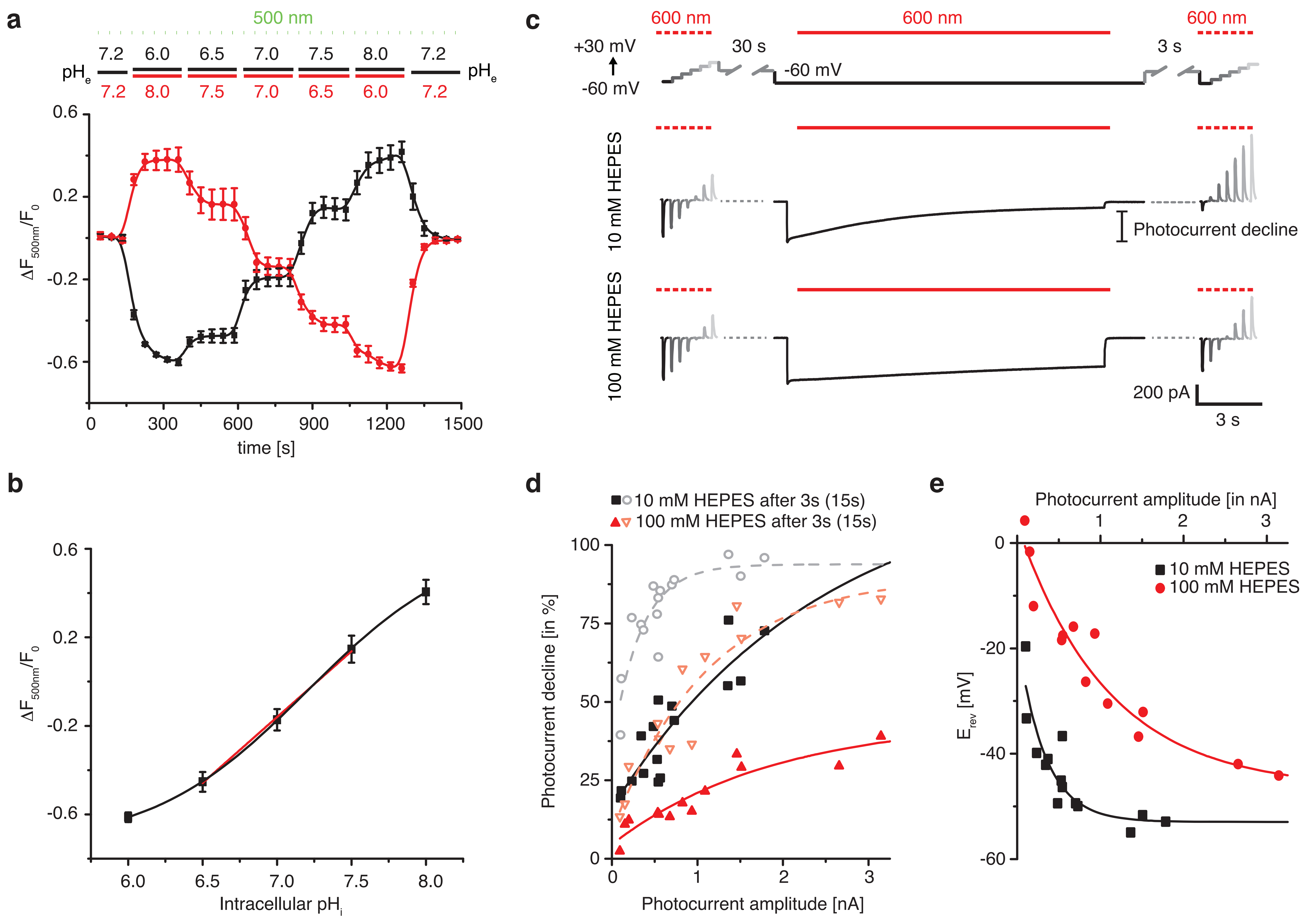

Supplementary Figure S4: Intracellular calibration of BCECF and slow photocurrent decline (a) Normalized fluorescence intensities of intracellular BCECF in HEK cells after excitation at 500 nm at different intracellular pH levels. HEK293 cells were treated with nigericin and the pH was adjusted using different extracellular solutions containing 135 mM KCl and the designated proton concentration (black: increasing pH value, n=11 cells; red: decreasing pH values, n=6 cells). **(b)** Dose-response curve (black) and linear approximation (red) of normalized BCECF fluorescence intensities after excitation at 500 nm at different intracellular pH levels (mean ± SD, n=17 cells) **(c)** Representative photocurrent traces at pH_e 7.2 with 10 mM or 100 mM intracellular HEPES. Reversal potential of Chrimson was probed 30 s before and 3 s after prolonged illumination at -60 mV. **(d)** Photocurrent decline 3 s or 15 s after the onset of 600 nm illumination at -60 mV with 10 mM (black/gray) and 100 mM intracellular HEPES (red/orange), depending on the peak photocurrent amplitude (exponential fits for better visualization). **(e)** Reversal potential measured 3 s after prolonged illumination at 600 nm with 10 mM (black) or 100 mM intracellular HEPES (red) in dependence on the peak photocurrent amplitude of the corresponding cell (exponential fits for better visualization).

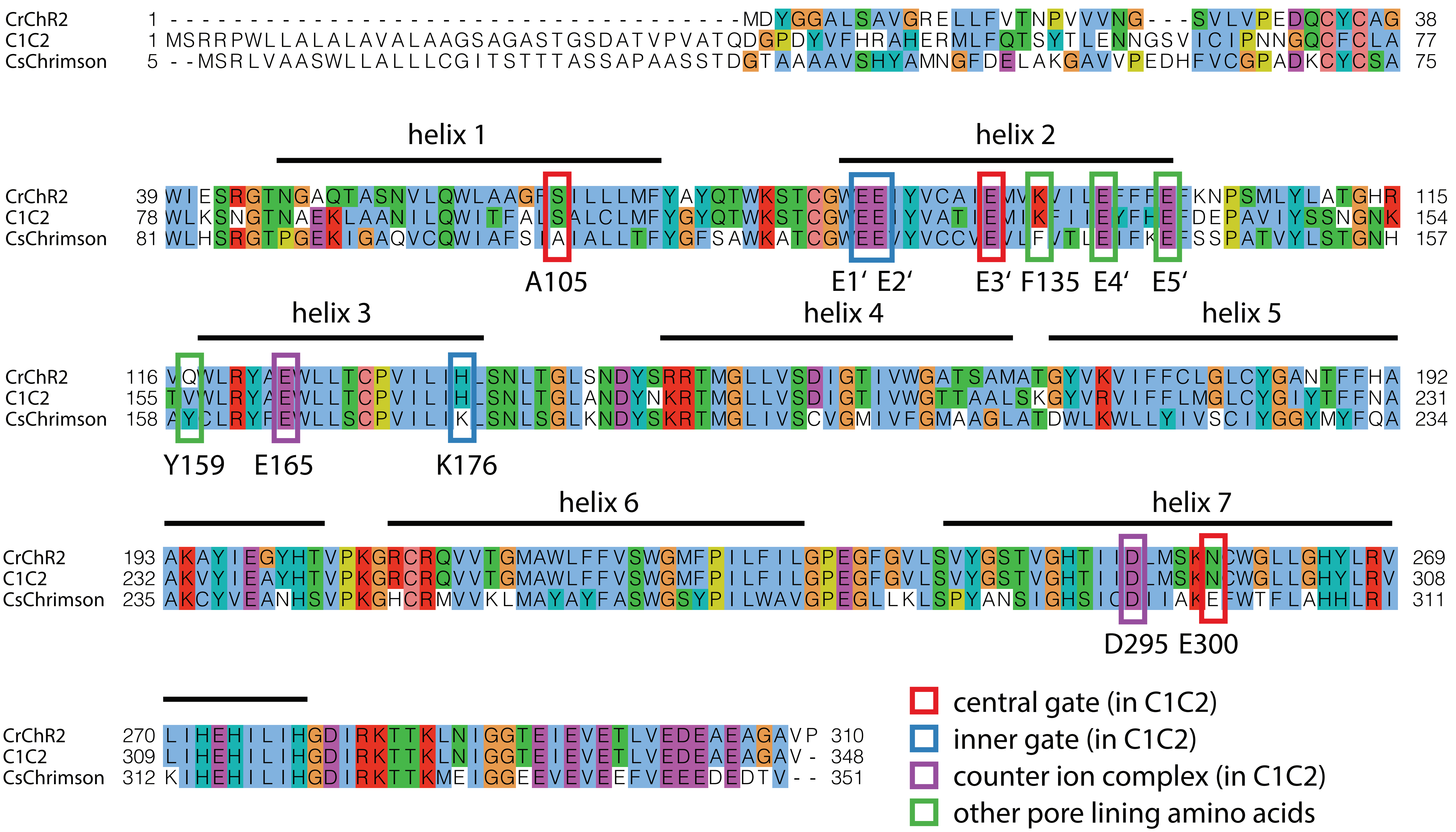

Supplementary Figure S5: Sequence alignment of CrChR2, C1C2 and CsChrimson

Color-coded frames indicate the mutated amino acid and their structural position in the C1C2 structure.

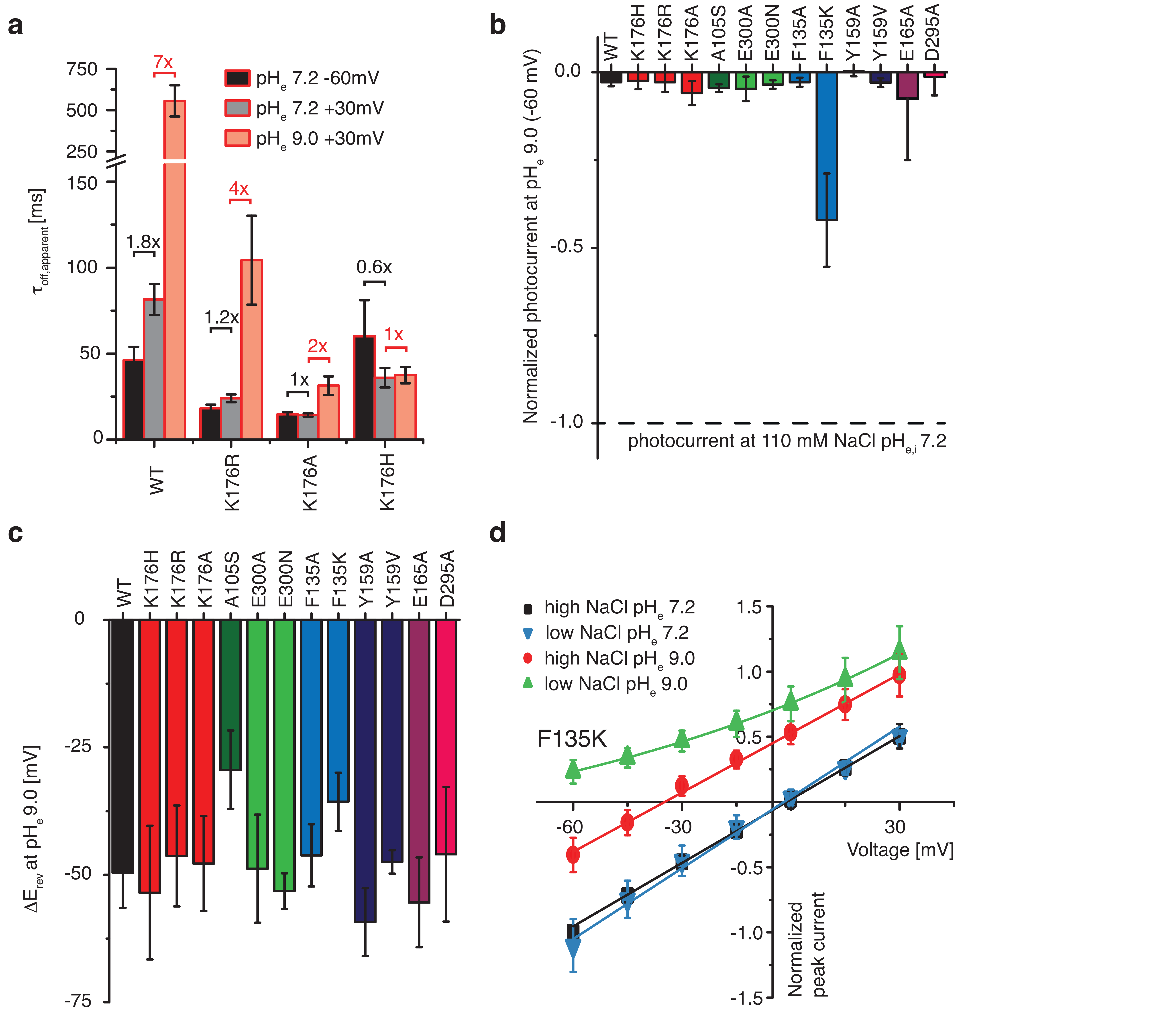

Supplementary Figure S6: Kinetics and proton selectivity of Chrimson pore mutants (a) Apparent off-kinetics of K176 mutants ($\tau_{apparent,\ off}$) at positive and negative voltages and pH_e 7.2 and pH_e 9.0. The voltage and pH dependency ($R_{Voltage} = \tau_{apparent}$(pH7.2,+30 mV)/ $\tau_{apparent}$(pH7.2,-60 mV), $R_{pH}=\tau_{apparent}$(pH9.0,+30 mV)/ $\tau_{apparent}$ (pH7.2,+30 mV)) of the depicted kinetics are shown in black and red numbers above the bars (mean ± SD; WT n=12-18 cells; K176R n=6-7; K176H n=5-9; K176A n=6-8). **(b)** Normalized photocurrent amplitudes at -60 mV and symmetric 110 mM NaCl and extracellular pH_e 9 and intracellular pH_i 7.2 (mean ± SD), photocurrent amplitudes were normalized to photocurrents at extracellular 110 mM NaCl and pH_e 7.2 (dashed line) and extrapolated to -60 mV after LJP correction; WT n=10 cells; K176H n=5; K176R n=8; K176A n=6; A105S n=8; E300A n=6; E300N n=6; F135A=11; F135K=11; Y159A n=6; Y159V n=6; E165A n=4; D295A n=4). **(c)** Reversal potential shifts upon exchanging the extracellular buffer from 110 mM NaCl and pH_e 7.2 to 110 mM NaCl pH_e 9.0 (mean ± SD, LJP corrected, WT n=10 cells; K176H n=5; K176R n=8; K176A n=6; A105S n=8; E300A n=6; E300N n=6; F135A=11; F135K=11; Y159A n=6; Y159V n=6; E165A n=4; D295A n=4). **(d)** The current-voltage dependence of normalized F135K peak photocurrents with different extracellular solutions of 110 mM NaCl and pH_e 7.2, 1 mM NaCl and pH_e 7.2, 110 mM NaCl and pH_e 9.0 and 1 mM NaCl and pH_e 9.0 and intracellular 110 mM NaCl and pH_i 7.2 (mean ± SD; n=6-18 cells).

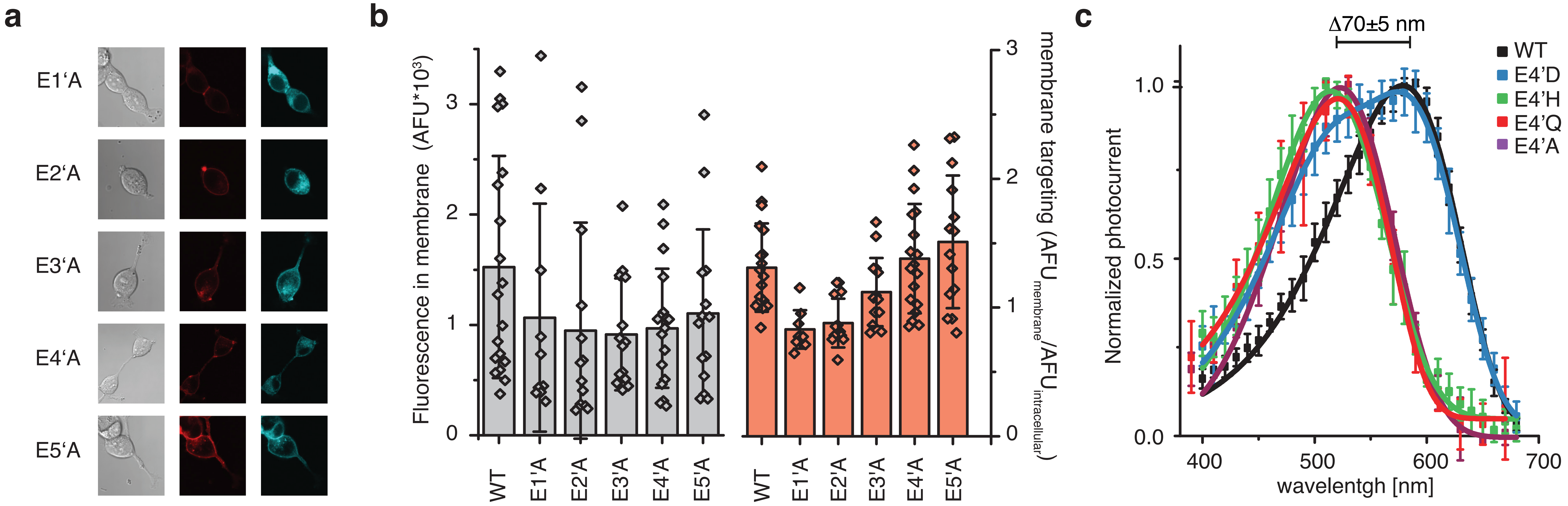

Supplementary Figure S7: Membrane expression of Chrimson Helix2 glutamate mutants E1'A to E5'A and action spectra of E4' mutants (a) Confocal microscopy of CsChrimson mutants in HEK293 cells under white light, excitation of R18 at 559 nm and excitation of mCerulean3.0 at 440 nm. **(b)** Fluorescence of mCerulean3.0 in the membrane (left) and membrane targeting (right) of Chrimson glutamate mutants (mean ± SD, WT n=19 cells, E1'A n=10 cells, E2'A n=14 cells, E3'A n=14 cells, E4'A n=18 cells, E5'A n=14 cells). **(c)** Normalized peak photocurrents after 10 ms excitation at different wavelengths of equal photon count (mean ± SD; symmetric 110 mM NaCl, $pH_{e,i}$ 7.2 and -60 mV; WT n=10 cells; E4'D n=10; E4'H n=5; E4'Q n=5 and E4'A n=6).
